# Supplementary figures and images for: High susceptibility of Tetranychus merganser (Acari: Tetranychidae), an emergent pest of the tropical crop Carica papaya, towards Metarhizium anisopliae s.l. and Beauveria bassiana strains
Source: PeerJ. 2022 Oct 24;10:e14064. doi: 10.7717/peerj.14064 (PMC9610662; doi:10.7717/peerj.14064)

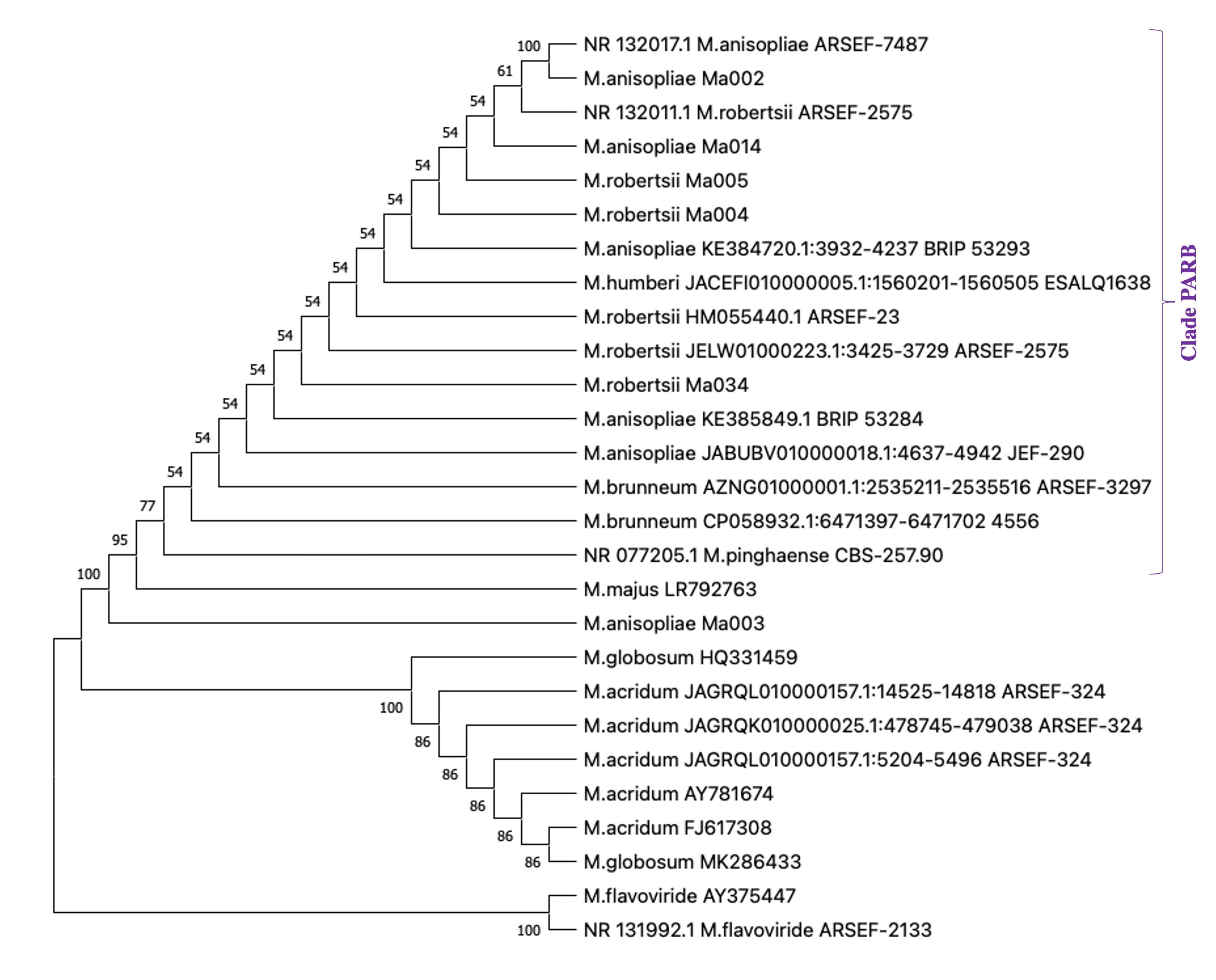

Supplement: Supplemental Information 4 — The ITS sequences of the most virulent M. anisopliae s.l. strains against T. merganser were compared with the ITS sequences of the Metarhizium reference strains reported in NCBI. The alignment by Clustal Omega (1.2.4) was used to construct a Phylogenetic tree with MEGA version X using UPGMA method. The bootstrap values represent the percentage of 1,000 replicates [file peerj-10-14064-s004.png]

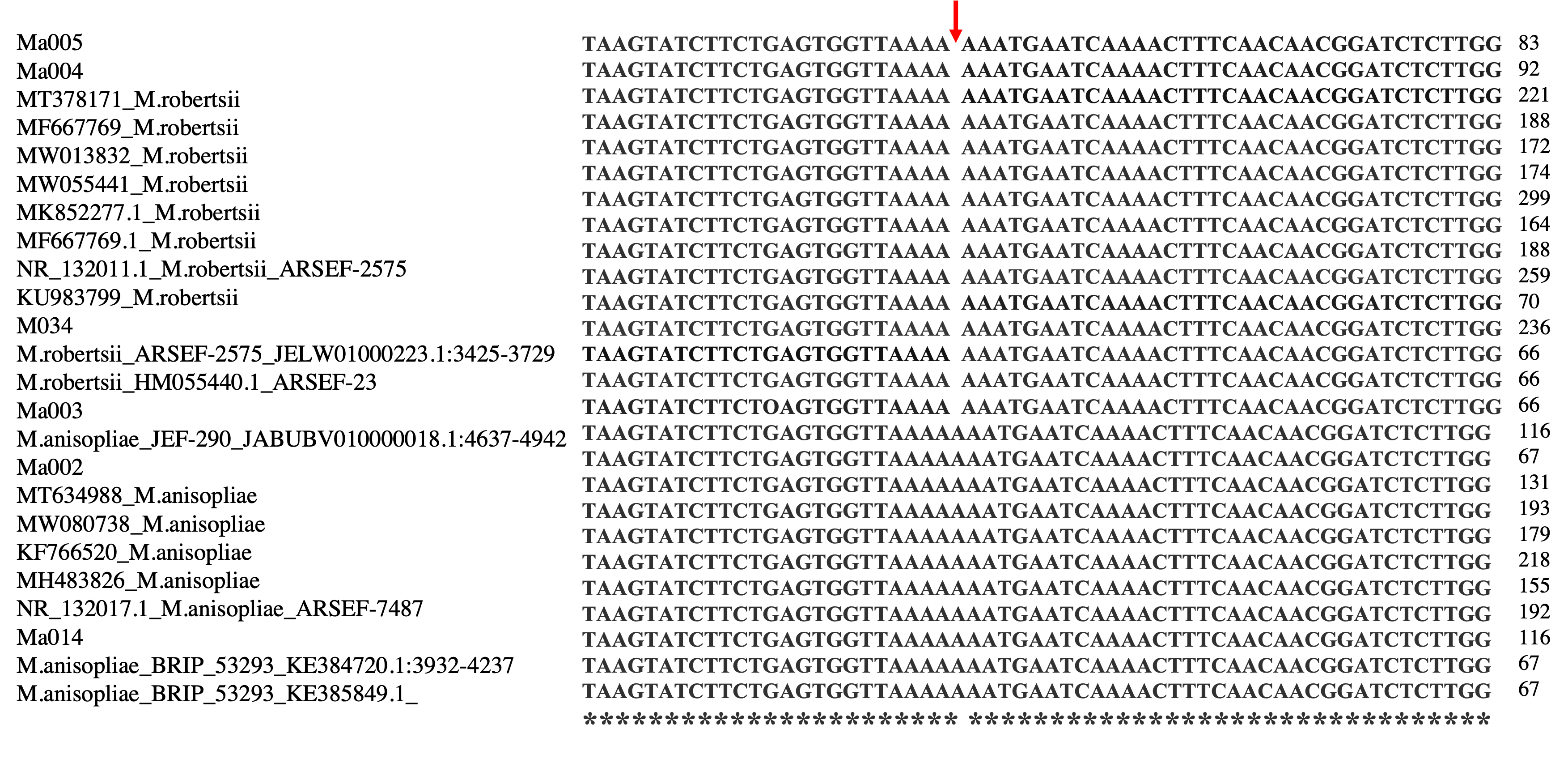

Supplement: Supplemental Information 5 — The alignment by Clustal Omega (1.2.4) shows a difference in the number of adenines between the ITS sequences of M. robertsii and M. anisopliae at position 28–35 of the reference strain of M. anisopliae JEF-290 (red arrow). [file peerj-10-14064-s005.png]
